# Understanding media empowerment: citizen journalism in Palestine

**DOI:** 10.1057/s41599-023-01526-z

**Published:** 2023-01-24

**Authors:** Ibrahim Horoub

**Affiliations:** Girne American University, Girne, Cyprus

**Keywords:** Cultural and media studies, Cultural and media studies

## Abstract

This study addresses the nature of citizen journalism in Palestine, with the chief aim of identifying its role in promoting professionalism, ethics, and objectivity, and empowering digital media, information accessibility, and democratization of media production. To this end, this study uses data from different universities across Palestine obtained through surveys that address digital media empowerment and non-professional users in the new media landscape. The study sample used was selected using the stratified sampling method. Upon completion of a pilot test, a total of 300 questionnaires were distributed among undergraduates of 10 universities across Palestine based on their willingness to participate and availability. Our results suggest that official media outlets can benefit from citizen journalism by providing accurate and valid sources of information to citizen journalists to increase engagement among their audiences as a result of enhanced perceptions of independency and credibility. However, our findings also reveal that citizen journalism does not make a significant difference in undermining gatekeeping practices over new media content in Palestine. Moreover, the results show that there is no relationship or correlation between the idea of adopting citizen journalism as a complementary method to traditional journalism and the credibility and reliability of news stories presented by citizen journalists.

## Introduction

Advancements in technology and social media have shown a significant impact on journalism as a profession, education, and at the higher education level across various disciplines (e.g. psychology, economics, technology, sociology, communication, marketing, and politics) (Aldaihani and Shin, 2022; Gorwa and Guilbeault, 2020; Loizzo et al., 2018; Mutsvairo and Salgado, 2022; McIntyre and Gyldensted, 2018; Zahoor and Sadiq, 2021). In this context, citizen journalism is focused on the current study that pertains to the equal amount of informational resources that are available for journalists as professionals and other people through social media and internet outlets (Allan, 2013). As the number of citizens spreading and creating news has drastically increased in recent years, the need for comprehending perceptions, influences, and professional aspects has become more prevalent. Literature shows a scarcity in this regard, and the gap is more clear in the context of Middle Eastern societies especially, Palestine. Noting the political situation and conflicts as well as constraints on the media, this study addresses the nature of citizen journalism practices in Palestine. It aims to identify the role of citizen journalism in the fight for professionalism, ethics, and objectivity, empowering digital media, information accessibility, and democratization of media production.

Citizen journalism studies have not been adequately addressed by communication scholars compared to other studies and articles that investigate traditional media. While there are several studies examining different aspects of citizen journalism (e.g. Goyanes, 2020; Salaudeen, 2021), there is a gap in the literature regarding professional aspects that have effects on the generated content by citizen journalists. In this regard, there is a vivid scarcity in the region of the Middle East and particularly, Palestine due to its special political and social issues. Notably, there have been several studies that assessed similar contexts focusing on conflict-related matters (e.g. Durante and Zhuravskaya, 2018; Hoxha and Hanitzsch, 2018; Zahoor and Sadiq, 2021), thus, leaving many aspects unexamined. Addressing this gap, the current study tends to establish a solid link between citizen journalism and Media empowerment by systematically analyzing professional and nonprofessional practices in this context.

With the rise of using social media and internet sources as news outlets (Raza et al., 2021), the current research aims to investigate how citizen journalists as gatekeepers interpret and perceive key concepts of journalistic work such as ethics, objectivity, gatekeeping, verification, information accessibility, and accuracy. Through a rigorous review of the literature on the subject, this study addresses the gap of Palestine as the location as well as the need for a better understanding of professional and nonprofessional practices related to this context. In many cases, mass media scholars cannot find appropriate methodological and theoretical frameworks for analyzing online users’ behavior and changing attitudes when dealing with such journalistic practices (Borah, 2017, 2011; Neuman and Guggenheim, 2011). Moreover, these scholars do not agree on one perspective regarding citizen journalism in terms of definition, boundaries, and nature. Many of them tend to utilize different academic fields when it comes to citizen journalism such as psychology, sociology, economics, etc. This in turn makes the task more difficult to understand the limits of citizen journalism and reveal the true nature of such interaction between different online users (Naab and Sehl, 2016). We also find it difficult to identify key theories or approaches that address a particular phenomenon without revealing the dimensions or contexts related to the subject of the study. The phenomenon of citizen journalism is constantly changing due to the nature of the newly generated content, and therefore a deep understanding of this phenomenon requires a correct selection of the intended approach that is related to it. In the context of citizens as journalists, there should be a personal contribution or a creative effort to consider such practices as a form of citizen journalism. We all know that traditional media content is produced and disseminated by media institutions that have different regulations and different editorial policies. However, in citizen journalism, the generated content becomes personal or non-institutional (Radsch, 2016; Vickery and Wunsch-Vincent, 2007). Most amateurs produce, edit, and broadcast such content independently and without contracting with professional media organizations.

Citizen journalism is based on an important assumption or principle in which the media content presented to the public does not depend on official sources or institutions but rather is produced and reproduced individually and then passed through a non-linear process. Therefore, citizen journalism provides people with a wider diversity of voices and alternatives than news organizations and other media outlets could deliver. In this case, it is a dynamic process in which online users can communicate their messages and interpret different forms of content without facing bureaucratic barriers, regulations, editorial policies, and other demands that professional journalism work requires. It is important to highlight that there is a significant difference between citizen journalism and traditional media in terms of professionalism. Many users who produce and publish citizen journalism content are unprofessional, and known as amateurs; whereas in traditional media, one of the conditions is journalists should be professional or at least have previous experience with professional journalism (Allen and Thorsen, 2009; Singer et al., 2011). This distinction gives professional journalists much more advantages than amateurs have in terms of the level of influence, objectivity, and ability to mobilize public opinion at both internal and external levels.

In addition, many professional journalists don’t trust the content generated by citizen journalists because it often lacks accuracy, professionalism, and objectivity which has resulted in. Various challenges to accepting or using such content in their professional journalistic work. These challenges include privacy issues, national security, and libel laws, as well as the complexities of the editorial policy of their media organizations (Naab and Sehl, 2016; Vos et al., 2012). Media ethics is one of the most important issues on which a great debate is being raised among communication scholars. Some argue that amateurs who produce and publish digital content do not take into account digital media ethics. They might not have sufficient knowledge about the principles and standards related to professional journalistic work such as impartiality, objectivity, independence, balance, verification, credibility, and so on (Tolmie et al., 2017). This problem becomes more sophisticated in light of the absence of global standards and principles that clarify the philosophy of professional journalism as well as the absence of common ground to develop digital media ethics under the umbrella of freedom of speech, democracy, and pluralism (Ryan, 2001; Singer, 2007; Singer and Ashman, 2009; Ward, 2005).

Following what was mentioned above, it is necessary to shed light on the nature of the relationship, roles, connections, and non-professional practices related to citizen journalism that bind all users in one system. Strictly speaking, there is an essential need to determine the nature of the information that users share and generate through various citizen journalism platforms. This will certainly lead us to identify the power of the impact of the messages disseminated by different online users on both internal and external levels. It is also important to investigate to what extent citizen journalism has contributed to undermining gatekeeping and limiting the ability of media outlets to control the new content and messages presented to the public. We should understand the role or roles that the gatekeeper plays in setting the agenda and controlling media discourse at all levels (Harrison, 2010; Shoemaker and Vos, 2009). This systematic analysis leads us to different sets of assumptions related to the particularities of citizen journalism and the various contexts in which such media content is produced and reproduced. Hence, this study aims to identify the degree to which citizen journalism can affect objectivity, power relations, professionalism, editorial practices, and ethics involved in reporting news and storytelling.

### Citizen journalism In Palestine

Numerous scholars have used various definitions for citizen journalism in different contexts. However, it can be stated that while citizen journalism can take various forms, the definition addresses ordinary people providing news information through cell phones and social media (Allan, 2013; Allan and Peters, 2015a). In this respect, citizens carry the role of a journalist amid an event, crisis, or social and political occurrences. This has been increasing over the past years with a significant rise after the outbreak of the COVID-19 global pandemic (Raza et al., 2021). In other words, this refers to situations, where citizens make news using their phones and share it on social media (e.g. Twitter and Facebook) including social, political, and/or economic matters (e.g. protests, police-related issues, corruption, and social movements) (Aldaihani and Shin, 2022; Al-Shami, 2019; McIntyre and Gyldensted, 2018). In its traditional form, journalism is embedded in media through professionals in the field, while in recent years with the spread of smartphones and internet accessibility worldwide, communication of content has drastically changed as audiences can be reached on a global scale (Domingo et al., 2008). It is important to note that citizens, in the role of journalist have not acquired the necessary skills and knowledge for appropriately selecting, and implementing journalism rules in their content. This has led to various challenges that are posed towards the credibility of such content (Al-Shami, 2019).

In the context of citizens as journalists, both engagement and reciprocation aspects persist that pertain to the bond between a producer of news and their audience. This form of participation in society is relatively new, particularly in countries, where media is restricted and/or controlled more rigorously by the governing body. As traditional news outlets lose their value among people due to advancements in Information and Communication Technologies (ICT) (Wahl-Jorgensen and Hanitzsch, 2009), it is imperative that social, and psychological aspects in this context are examined so that professionals in this area can maintain their roles. As citizens can provide news in an instant using social media in a convenient manner, perceptions of news audiences have changed to a new normal, in which constant updates are expected (Allan and Peters, 2015b). Users of social media are prompted by routine information on a global scale simply via their smartphones, which has established an atmosphere, where ordinary citizens can act as journalists.

The increase in the extent to which ordinary citizens produce and spread news has been noticed in several studies (e.g. Carr et al., 2014; Durante and Zhuravskaya, 2018; Lin, 2014; Nah et al., 2017; Zahoor and Sadiq, 2021). However, the extant literature shows a certain gap regarding the linkage of psychological elements and subsequent societal impacts of citizen journalism. In this context, the perception of individuals regarding the *credibility* of citizen-produced news has been noted as an important element (Johnson and Wiedenbeck, 2009; Nah and Chun, 2012). Notably, skeptical and cynical individuals have been found to have a higher tendency to show trust toward news generated by citizens as opposed to official news (Carr et al., 2014). The influence of citizen journalism on society due to its psychological influences cannot be neglected (Salaudeen, 2021). This has become more prevalent due to the decreased level of *trust* in the mainstream media and that of official governmental outlets (Allan and Thorsen, 2009). It has been also noted that citizens with lower trust can be engaged with media and news through citizen journalism (Raza et al., 2021; Saragih and Harahap, 2020). In contrast, the credibility, and standards of such news have been under question and subject to criticism widely across the world. Due to the aforementioned contradictions and low level of trust as a psychological factor (Farmanesh and Zargar, 2021), the influences of citizen journalism on society can be limited and/or reduced (Carr et al., 2014).

Following what was mentioned above, the perception of the audience and the *quality* of information generated through citizen journalism are among the key aspects that influence media–society linkage. Moreover, the extent to which the information is *accurate* and is accepted or rejected by the audience is another influential aspect of the attitude of an audience as consumers (Lin, 2014; Nah and Chung, 2020). Trust and credibility, therefore, are imperative as psychological aspects that influence the attitude of the audience towards news, particularly those generated by other citizens. While the majority of studies addressing this notion have taken a survey or experiment approach (Carr et al., 2014; Mutsvairo and Salgado, 2022), the current research uses a similar approach to obtain in-depth data to fill another gap found in the literature. Regarding credibility and trust, there are important factors that can influence the perception of the audience such as source, content, and medium (Vraga et al., 2012). These factors are influential on health behaviors (Pornpitakpan, 2004; Raza et al., 2021), social matters (Kelman and Hovland, 1953; Goyanes, 2020), and brand-related attitudes (Goldsmith et al., 2000; Thomas and Sooknanan, 2019). In addition, bias in the presentation of the news, and low quality of content have a negative impact on the perception of credibility (Chung et al., 2020; Kiousis, 2001), while competence, timeliness, and dynamism can have both positive or negative impacts on the audiences’ perception (Chan-Olmsted and Cha, 2008; Gaby and Caren, 2012).

As the outbreak of COVID-19 vastly limited activities and forced online communications to incorporate all aspects of media, the behavior and attitude of people towards government and official reports were seen as bipolar and lacking consensus (Wu, 2020). Medical experts, activists, and official actors in the government and citizens alike were found to have generated news and content regarding the virus, its effects, and the trustworthiness of news outlets. Official sources’ endorsements did not have the same level of credibility perceived among the audience when compared to non-official ones. It was also noted that the perception of independent sources can have a positive impact on trust among critics of government and individuals without predispositions towards the source, implying that citizen journalists can positively impact health-related behaviors during a pandemic when the credibility of government and its performance is under question and is being challenged. This is beneficial for practitioners as they can delegate the distribution of news to citizen journalists, when it comes to technical matters in a society that is undergoing a crisis (e.g. vaccinations, statistics, and information regarding the virus) (Blair et al., 2017; Coppock and McClellan, 2019; Liao et al., 2010; Raza et al., 2021). Depending on the basis of the government and its degree of control over the society (democratic or authoritarian), trust can be hindered by official media outlets, leading to potential health hazards due to noncompliance (Wu, 2020). This further drives the current research to focus on trust and perception of credibility as crucial factors that can have significant impacts on society.

The aforementioned lack of trust can be seen more vividly among Middle Eastern nations due to the format of governing systems and limitations on the media in this region (Bakis and Karakoç, 2015; Bellin, 2004; Kazemi and Norton, 2006; Kurd, 2022). During the pandemic period, the source that can aid the government to implement its policies regarding health-related issues and safety is citizen journalists as they are recognized as independent and thus, are more trustworthy in society in terms of risk perception and communication (Wu, 2020). This becomes highly important for the Middle Eastern context as authoritarian regimes are dominant and therefore, need mediating elements (i.e. citizen journalists) to enhance trust and reduce credibility deficit via increased transparency of policies and regulations during crises (Chuang et al., 2015; Zoorob and Salemi, 2017).

Both individual and group settings can be used by citizens as journalists and social media, blogs, or websites can be their outlets, which often cooperate with professionals in media and journalism as well (Loizzo et al., 2018). Combining computer skills in journalism, sharing knowledge, humanitarian-related subjects, and community building have been noted to be the main drivers of citizen journalists. This shows that preferences and individual traits are more dominant for citizens to share news rather than professionalism. Within the context of communication, journalism deployed by citizens is regarded as positive, constructive, and solution-oriented, which varies from the general notion of journalists as professionals that mainly focus on negativity (e.g. incidents, crises, and issues). The positive psychology aspect can have important outcomes such as enhancing awareness and knowledge for the audience, establishing bonds between the audience and news provider, and increasing engagement towards the issue/subject at hand (Sheldon and King, 2001).

Compared to professional journalists, citizen journalism does not have the same level of accuracy, autonomy, and objectivity (de Zúñiga, 2013), which varies fundamentally in the perception of the audience (Nah et al., 2017). In this sense, several aspects impact the attitude of the audience towards news generated by citizens that are namely, expressions of individual preferences, coverage of content that is deemed necessary, higher connectedness and engagement with the audience, opinion-centered and less factual, and biased. These factors can have positive or negative impacts on the audience’s perception and attitude (Nah and Chung, 2012). This implies that citizens in the role of journalists focus mainly on engagement and not the validity or the process, which differs from the professional approach deployed by journalists. However, it has been noted that to enhance the engagement (perceived and actual) of the audience for a media outlet, the inclusion of citizens as gatekeepers seems to be a necessity.

Citizen journalism plays an influential role in the Palestinian community in which Palestinian users receive information, opinions, and attitudes from the new media platforms and they are largely affected by the generated content of citizen journalists especially when it comes to the Palestinian - Israeli conflict. Accordingly, new media coverage of political issues not only affects Palestinian’s attitudes but also activates their national identity and ethnic tendency. It is reasonable to state that new media platforms affect social and cultural backgrounds. New media play a significant role when covering problematic political issues. Therefore, in such cases, the conflict becomes a major news story for the public. This happens through the selection of news stories presented in new media platforms and re-building the Palestinian consciousness towards the existential conflict they face. On the other hand, new media platforms are less likely to play an independent role when nonprofessional journalists misrepresent the other group on new media platforms.

This has made it important to interpret how different users of new media platforms deal with such coverage. The central argumentation should focus on the political conditions and the legal environment that control the news stories and media content generated by citizen journalists. Those political activists on social media platforms try to create a negative image of the other group. This is done by providing the public with certain information while maintaining the positive image of their group. It can be said that political views adopted by nonprofessional journalists are different when dealing with the Palestinian- Israeli conflict. This has dramatically led to a state of contradiction and widened the gap between those opponents. Therefore, due to these complex political contexts related to the Palestinian–Israeli conflict, some new media platforms try to impose many restrictions on publishing Palestinian content, and this contributes significantly to promoting gatekeeping practices on the one hand and it affects freedom of expression on the other. The Palestinian content disseminated on new media platforms plays an essential role in persuading the Palestinian guys to act in certain ways especially when there are potential clashes with the Israeli forces. In every crisis or revolutionary wave, the debate is raised about the Palestinian media discourse and its power in addressing such events. The debate is also raised about the persuasion methods and techniques that Palestinian activists employ in promoting national discourse and mobilizing public opinion locally and internationally.

## Theoretical setting

The notions of citizen journalism, technology, empowerment (Cervi and Marín-Lladó, 2022), and social movements are complex; and several theories can be linked to the current context. However, as the research focuses on professional aspects that have effects on the generated content by citizen journalists, the following concepts are deemed fit for achieving the research objectives.

The gatekeeping theory is embedded in the premises of the current research as it is commonly used to address (a) constant occurrences of events in a Spatio-temporal context, and (b) limitations of official media in terms of coverage. Hence, a selection process is conducted to generate news that is worthy to be shared and is referred to as the gate with the individual responsible for selection known as the keeper (Shoemaker and Vos, 2009). Through this conception, this research looks into the influences of citizen journalists on psychological factors important in the society through usage of ICT (e.g. social media platforms, and smartphones). In this respect, speed and level of access are essential as audiences are equipped with the internet that enables them to read worldwide news instantly. In the process of gatekeeping various stages are involved that is, writing, production, and content management. Notably, professional journalists use group settings that delegate gatekeeping processes.

The selection process is derived from the level of attraction or interest in the topic, its vitality for society and/or the audience, and the visual elements and their effects that attract the audience (Cuillier, 2012). This theory pertains to the fact that individual traits are not dominant among professional journalists for the creation of news but rather subconscious psychological elements are prime in the decision-making process. An example can be seen through the premise of terror management theory, where journalists may show bias unknowingly regarding reports of death or dire situations (e.g. COVID-19 reports). This is based on the perception of news providers as they can include their cultural or personal views in what they report. In this context, political ideologies, personal backgrounds, socialization, and norms have been noted to be influential psychological factors. Linked to framing theory, social constructions are conveyed by journalists that incorporate their values, ideas, and professional behaviors, and are portrayed through frames of the story (D’Angelo, 2019).

The theory of collective behavior that incorporates behaviors such as crowd, mob, panic, riot, disaster behavior, rumor, mass hysteria, moral panic, and crazes is a relevant construct that is linked to psychological and sociological elements in society provoked by news and media (Aguirre et al., 1998; Drury and Reicher, 2020). This relates to large-scale activities that are not fully organized but are engaged by a considerable number of people. In the aftermath of a crisis (i.e. COVID-19 pandemic), collective behavior has become more vivid as social connectedness and health behaviors as well as risk communication became prevalent and influenced the psychology of crowds (Templeton, 2021). Qualitative methodology and ethnography are among the commonly used approaches to understanding the psychological and social elements in this regard (D’Angelo, 2019).

Again the cognitive component is vital in forming attitudes and behaviors toward news. In this respect, availability and applicability are noted to be the key factors that are derived from the expectancy-value theory (Chong and Druckman, 2007) and combined with the cognitive definition of accessibility. Based on available knowledge of an individual regarding an event or problem, judgments that are made can yield being in favor or against the portrayed in the news. This can in turn lead to the formation of new beliefs, contradictions, and/or approval of existing beliefs (D’Angelo, 2019).

The current study tends to provide an in-depth understanding of professional and nonprofessional practices linked to citizen journalists and their influence on society through the eyes of citizen journalists as gatekeepers of news. The literature shows social, psychological, political, and economic elements such as trust, risk, health, justice, and civic aspects as dominant factors, which the current study tends to shed light on and highlight their importance (e.g. Aldaihani and Shin, 2022; Browning et al., 2021; Miller, 2019; Nah and Yamamoto, 2019; Nah and Chung, 2020; Raza et al., 2021; Salaudeen, 2021; Wall, 2018). Importantly, due to the high level of corruption among Arab countries (Al-Shami, 2019), particularly Palestine (Badwan and Atta, 2019; Schoeberlein, 2019), this research expects to note politics-related elements that affect the perception and attitude of people in the society, when engaged with news (i.e. citizen journalists) that are representations of low trust levels.

## Methodology

### Sampling method and data collection

Because the sampling method and data collection are the original part of this research, especially concerning the primary data which is represented by the standardized questionnaire, great efforts have been made to verify the reliability of the collected data, the accuracy and validity of the selected procedure and the representativeness of the research sample. Therefore, the self-administered questionnaire for this study was designed using relevant, valid, and available scales based on the reviewed literature. A pilot test was deployed with 20 participants to examine the readability and validity of the scales’ items. The pilot testing has been conducted to improve the structured questionnaire by choosing a group of participants to be the sample of the pilot test before adopting the main version of the current questionnaire. Upon completion of the pilot test, a total of 300 questionnaires were distributed among undergraduates of 10 universities across Palestine based on their willingness to participate and availability. All questions were designed on a 5-item Likert scale ranging from 1 = totally disagree, to 5 = totally agree.

To ensure ethical means of conduct several aspects were taken into consideration that are namely, each participant was informed of research purposes and objectives; a written consent form was provided to participants; withdrawal was made possible at any stage; no personal information was collected and confidentiality was given to participants; and original data was deleted upon computerization of responses. These measures ensured compliance with ethics while reducing the rate of common method bias.

Furthermore, the sample of this current research was selected by using the stratified sampling method. This is important to avoid bias and other negative effects on the procedures of this current research by giving each case of the population an equal chance of being selected. It should be noted that we use stratified sampling because we deal with a large amount of data.

The researcher has selected a representative sample of the targeted population to examine the hypotheses of this study. Therefore, 300 undergraduates from local universities in the West Bank in Palestine were selected to be the sample size. Most students are supposed to be active users of social media platforms. They represent different backgrounds, different regions, and different affiliations. These students were given a questionnaire because they represent reliable cases that can be tested and verified.

### Method of data analysis

The analytical survey is one of the advanced statistical techniques to analyze large amounts of data which can be used in all areas of social research, especially mass media research. However, it cannot be used as a tool for data analysis separated from the theoretical and methodological bases. The SPSS software was used to analyze the obtained data through specific criteria that fit the deployed analytical technique of this research. This technique is deemed appropriate for the current study as it entails a latent variable, a small sample size that requires statistical power, and does not concern the normality of distribution in the data.

To test the hypotheses, three types of statistical tests were adopted to get accurate results. The One-way ANOVA was used to test hypothesis 1 whereas Two-way ANOVA was utilized to test hypothesis 2. The Chi-Square Test of Association was used to examine hypothesis 3.

A one-way ANOVA test was used to examine if there is a statistically significant difference in the ability of citizen journalism to undermine gatekeeping practices over media content produced by Palestinian citizen journalists based on exercising a form of censorship over the content they disseminate. The respondents of the current questionnaire had to rate it on a scale from 0 to 10 if citizen journalism can undermine or weaken social and political gatekeeping control over media content. A larger number indicates a higher level of undermining. They also had to select the degree of agreement on whether social media platforms that are popular in Palestine could exercise a form of censorship over the content that users provide or publish. The second hypothesis used a Two-way ANOVA test to check if there is no significant difference in the effect size of citizen journalism on freedom of speech and information accessibility based on citizen journalism’s lack of accuracy, professionalism, regulation, objectivity, verification, and digital media ethics. One dependent variable which includes information accessibility and freedom of speech is on scale level in addition to two independent variables (each one has many levels) which include accuracy, professionalism, regulation, objectivity, verification, and digital media ethics related to citizen journalism in Palestine. The Chi-Square Test of Association was used to find the association and relationship between the two categorical variables which include citizen journalism as being a complementary method to traditional media and the reliability of news stories presented by citizen journalists in Palestine. Table 1 shows the three types of statistical tests which were exploited to inspect the hypotheses of this current study.

**Table 1:** Types of statistical tests.

Hypotheses	Type of statistical tests by (SPSS)
Hypothesis 1	One-way ANOVA
Hypothesis 2	Two-way ANOVA
Hypothesis 3	Chi-Square Test of Association

It should be pointed out here that the researcher manipulated the independent and dependent variables to build good relationships and make significant comparisons between different contexts related to citizen journalism. The demographic factors included in the survey (i.e. age, gender, and education) were controlled in the analysis based on their impact on citizen journalism. These measures were undertaken based on the extant literature and relevant studies in the same context. This analytical framework has contributed significantly to obtaining more accurate results. Utilizing the current variables and building clear and direct hypotheses have made it possible to overcome the bias that might affect the objectivity of the research, and thus we can get a better understanding of the characteristic features of citizen journalism practices in Palestine.

### The analysis

The results of this quantitative study highlight the statistical analysis procedures by examining the current hypotheses and variables based on the methodology adopted in this study. Therefore, the researcher proposes the hypothesis at the beginning and then examines it based on the adopted analytical framework. The researcher also provides an explanation and a deeper understanding of the contexts, connections, and relationships related to the intended hypothesis.

#### Hypothesis (1)

There is a statistically significant difference in the ability of citizen journalism to undermine gatekeeping practices over media content based on exercising a form of censorship over the content users disseminate.

The One-way ANOVA test was used to examine this hypothesis. The results showed that *P* value (0.549) > alpha (0.05), Therefore, we failed to reject the null hypothesis; there was not enough evidence for a significant difference in the ability of citizen journalism to undermine gatekeeping practices over media content based on exercising a form of censorship by social media platforms over the content users disseminate. This mostly occurs due to the various publishing restrictions that new media platforms impose on some Palestinian users. Tables 2 and 3 show the results of the current hypothesis by using SPSS analysis.

**Table 2:** Descriptive (undermine gatekeeping practices).

	*N*	Mean	Std. deviation	95% Confidence interval for mean		Minimum	Maximum
				Lower bound	Upper bound		
Strongly disagree	5	6.6000	2.96648	2.9166	10.2834	2.00	10.00
Somewhat disagree	37	6.6486	2.17583	5.9232	7.3741	1.00	10.00
Neither disagree or agree	56	5.8036	2.58260	5.1119	6.4952	1.00	10.00
Somewhat agree	94	6.1596	2.10122	5.7292	6.5899	1.00	10.00
Strongly agree	108	6.1296	2.47263	5.6580	6.6013	1.00	10.00
Total	300	6.1500	2.35215	5.8828	6.4172	1.00	10.00

**Table 3:** ANOVA Undermine gatekeeping practices.

	Sum of squares	df	Mean square	*F*	Sig.
Between groups	16.987	4	4.247	.765	.549
Within groups	1637.263	295	5.550		
Total	1654.250	299			

To clarify this phenomenon, several factors that directly contribute to the inability of new media platforms to control new media content should be taken into consideration. First, the nature of the content generated by Palestinian users on the Internet cannot be measured in terms of its effect size without the direct interaction of users with it. In most cases, poor content that users do not interact with on the Internet is ignored by different power structures because it does not represent a direct threat to the attitudes and ideologies of the target audience. It is also difficult to implement the censorship system on the newly generated content and new media platforms because these new platforms are not directly linked to official institutions controlled by governments and different power structures.

Because the study sample comprises communication students in different Palestinian universities, this phenomenon can also be explained by analyzing the political and social contexts that directly affect Palestinian users. Citizen journalism plays an influential role in controlling the Palestinian society in which Palestinian students receive information, opinions, and attitudes from the new media platforms and they are largely affected by adopting new perspectives, interpretations, and perceptions about the Palestinian - Israeli conflict. However, this requires a democratic political climate and constitutional sovereignty that guarantees their rights and duties as well as liberating media institutions from classical ideological hegemony imposed by different power structures in the Palestinian community. There is a tendency among the study respondents that citizen journalism can undermine social and political gatekeeping control over media content. This is related to their belief that citizen journalism plays a significant role in the fight for information accessibility and freedom of speech. Information accessibility is the right to obtain, receive, and use data to exploit it for personal and institutional purposes. Laws and constitutions around the world guarantee the right of citizens to access information unless such information violates national security and institutional interests. This active role that citizen journalism plays in the fight for freedom of expression in Palestine has become more important in light of the various restrictions and complexities that negatively affect traditional media in terms of controlling the content and mobilizing the audience.

68% of respondents (Participants who somewhat agree plus strongly agree) believe that through citizen journalism individuals can create a kind of distinctive identity through which they reflect their opinions, attitudes, and perspectives. This is basically because content production is no longer limited to institutions and individuals working in the field of mass communication. The public has become active in the process of exchanging information and building news stories alike.

#### Hypothesis (2)

There is no significant difference in the effect size of citizen journalism on freedom of speech and information accessibility based on citizen journalism’s lack of accuracy, professionalism, regulation, objectivity, verification, and digital media ethics. The Two-way ANOVA test was used to examine this hypothesis. The results showed that *P* value (0.109) > alpha (0.05), so we failed to reject the null hypothesis and there was not enough evidence for a significant difference in the effect size of citizen journalism on freedom of speech and information accessibility based on citizen journalism’s lack of accuracy, professionalism, regulation, objectivity, verification, and media ethics. Table 4 shows the results of the current hypothesis by using SPSS analysis.

**Table 4:** Tests of between-subjects effects. Dependent Variable: information accessibility and freedom of speech.

Source	Type III sum of squares	df	Mean square	*F*	Sig.	Partial eta squared
Corrected model	93.388^a^	24	3.891	1.424	.094	.111
Intercept	8302.563	1	8302.563	3037.760	.000	.917
Lack of objectivity						
Verification credibility	20.002	4	5.000	1.830	.123	.026
Media ethics						
accuracy						
professionalism	18.582	4	4.646	1.700	.150	.024
regulation						
*Lack of objectivity*						
verification credibility						
media ethics * accuracy	64.381	16	4.024	1.472	.109	.079
professionalism						
regulation						
Error	751.608	275	2.733			
Total	18463.000	300				
Corrected total	844.997	299				

^a^*R* squared = 0.111 (adjusted *R* squared = 0.033).

In many cases, the new media content disseminated by users on the internet lacks accuracy, regulation, and professionalism. However, these contexts do not affect the margin of freedom available to them, as they can express their different opinions and attitudes, and they can easily access different information and data. This in turn does not affect the power of citizen journalism in comparison to traditional media outlets.

Therefore, it is reasonable to assume that citizen journalism plays a significant role in the fight for information accessibility, human rights, and democracy. This is also clear when it comes to information accessibility and the rights to obtain, receive and use data to exploit it for personal and institutional purposes. It is observed that citizen journalism helps to reveal certain facts related to the Palestinian–Israeli conflict that do not appear in the traditional media, and this in turn contributes greatly to the democratization of the content and helps people to obtain information and facts easily. However, most journalistic practices adopted by different users and amateurs on the Internet lack ethics and objectivity when it comes to reporting and storytelling. These amateurs do not often check the sources from which they take the news, yet most of them claim professionalism and objectivity. This negatively affects the popularity of citizen journalism and increases the possibility of fabricating news stories and reports to obtain more personal interests and benefits.

Most participants are aware that citizen journalism affects objectivity and ethics involved in reporting news compared with traditional media in Palestine. This is because the new media content lacks professionalism and in many cases, it reflects personal opinions, unlike traditional journalism, which reflects the editorial policy and ideology adopted by the media institution. In addition, citizen journalism practices in Palestine lack ethics and regulation due to the absence of a digital ethical framework that protects and drives online journalistic practices. This orientation comes from the belief that any journalistic work, including citizen journalism, must operate under the umbrella of ethics, credibility, and regulations.

However, most participants believe that citizen journalism plays a significant role in the fight for information accessibility, human rights, and democracy. This is clear when it comes to information accessibility and the rights to obtain, receive and use data to exploit it for both personal and institutional purposes.

#### Hypothesis (3)

There is a relationship between citizen journalism as being a complementary method to traditional media and the reliability of news stories presented by citizen journalists.

The Pearson Chi-Square test showed that *P* value (0.860) > alpha (0.05), that’s why we failed to reject the null hypothesis and there was not enough evidence for a significant relationship between citizen journalism as being a complementary method to traditional media and the reliability of news stories presented by citizen journalists. Most journalists and users who take advantage of the generated content on the Internet believe that the reliability of the news presented does not directly affect professional practices when adopting citizen journalism as a complementary method or an alternative option to traditional journalism. This belief is related to the circumstances and contexts surrounding citizen journalism. Most amateurs who present themselves as journalists do not take into account the rules and standards that govern professional journalism practices. Therefore, there is no relationship or correlation between the idea of adopting citizen journalism as a complementary method to traditional journalism and its credibility and reliability of it. Tables 5 and 6 show the results of the current hypothesis by using SPSS analysis.

**Table 5:** Chi-Square tests.

	Value	df	Asymp. Sig. (2-sided)
Pearson Chi-square	3.964^a^	8	.860
Likelihood ratio	3.573	8	.893
Linear-by-linear association	.854	1	.355
No. of valid cases	300		

^a^6 cells (40.0%) have expected count <5. The minimum expected count is 0.08.

**Table 6:** Case Processing Summary.

	Cases					
	Valid		Missing		Total	
	*N*	Percent	*N*	Percent	*N*	Percent
Complementary method* reliability of news stories	300	100.0	0	0.0	300	100.0

Most of the respondents in the survey could not strongly agree or strongly disagree about whether they can trust the reliability of news stories presented by citizen journalists. This reflects the debate over citizen journalism on the one hand and reflects the different opinions about the nature of the content that should be adopted by citizen journalists and its characteristic features. Most of the professional journalists who are active in the official Palestinian media sector rely to a considerable degree on the content generated through new media platforms. However, these journalists do not always check the reliability of news stories when they quote reports and statements from nonprofessional journalists. Palestinian activists are aware of the importance of social networks in mobilizing public opinion and this motivates them to adopt some news stories without testing the credibility and reliability of the new content presented to the audience. Therefore, it can be said that the adoption of citizen journalism as a complementary approach to traditional media is not related to whether the adopted content is reliable or not. It is mainly due to the factors and circumstances surrounding the production and reproduction of news stories.

27% of the respondents (Participants who somewhat agree plus strongly agree) believe that citizen journalism does not lack regulation, verification, and objectivity. This is fundamentally related to their perception of citizen journalism that it can be considered a complementary method to traditional media. However, 47.3% of respondents (Participants who somewhat disagree plus strongly disagree) confirm that citizen journalism lacks regulation, verification, and objectivity.

Another factor that significantly contributes to the development of citizen journalism is the diversity of sources and the preservation of some characteristics that we do not find in traditional media, such as exclusivity and quick feedback. 65% of participants in the current questionnaire responded that citizen journalism is better than traditional journalism in terms of diversity of sources and exclusivity. This is important to increase the reliability of news stories presented by citizen journalists and it also serves the effect size of citizen journalism on the target audience.

## Conclusion

There is a real need to understand citizen journalism and the nature of its generated content presented to the public across new media platforms. This is important because understanding citizen journalism and analyzing the different contexts in which it operates will contribute to the development of new methods and models of mass communication on the one hand, and will certainly promote democratic and liberal practices in postmodern societies on the other hand. Therefore, the quality and nature of the content that users provide through new media platforms can be identified by understanding and analyzing the various factors and variables that affect citizen journalism, such as professionalism, accuracy, credibility, gatekeeping practices, digital media ethics, validity, verification, and objectivity.

This study indicates a set of conclusions. Some of them relate to the generated content by Palestinian citizen journalists such as the reliability and accuracy of news stories presented by citizen journalists and other conclusions relate to the journalistic practices that directly impact citizen journalism and its effect size on traditional media such as information accessibility, regulation, verification, media ethics, and gatekeeping practices. The results of this quantitative research have highlighted the statistical analysis procedures by examining the scientific hypotheses and variables based on the methodology adopted in this research.

Notably, citizen journalism operates within complex contexts that cannot be controlled in most cases, this in turn makes it difficult to compare such generated content with professional media content produced and reproduced by traditional media. In many cases, the content generated by citizen journalists on the internet lacks accuracy, regulation, and professionalism. However, these practical inconsistencies related to citizen journalism do not significantly affect the margin of freedom available to citizen journalists, as they can express their different opinions and attitudes, and they can easily access different information and data. The statistical analysis also reveals that most professional journalists who take advantage of citizen journalism’s content on the Internet believe that the reliability and credibility of the new media content presented by citizen journalists do not directly affect professional practices when adopting citizen journalism as a complementary method or an alternative option to traditional journalism.

This shift towards new media platforms by traditional media outlets implies that the reliability of the newly generated content does not significantly impact such professional journalistic practices. It is mainly due to the factors and circumstances surrounding the production and reproduction of news stories. This phenomenon in which professional journalists do not always check the reliability of news stories produced by citizen journalists when they quote reports and statements from nonprofessional journalists becomes more popular on traditional media platforms when there is a lack of sources and when the scoop (a news report that is reported first by one news organization) becomes a priority for workers in mass media outlets.

Finally, it may be concluded that any journalistic work, including citizen journalism, should operate under the umbrella of ethics, credibility, objectivity, accuracy, professionalism, and regulation. It is reasonable to say that the negative effects of citizen journalism can be avoided by adopting digital media ethics and developing rules and standards related to key practices such as objectivity and verification. Developing digital media ethics is important because the new media are based on the circulation of news stories via the Internet, and this should be consistent with the adopted ethics in traditional media, taking into account the nature of newly generated content and the different contexts that govern online journalistic practices. The active role that citizen journalism plays in the fight for freedom of expression has become more important in light of the various restrictions and complexities that negatively affect traditional media in terms of controlling the content and mobilizing public opinion. Therefore, the effectiveness of citizen journalism practices can be enhanced by improving professional practices and the adoption of digital media ethics by amateurs so that they can apply these standards to their online journalistic activities.

### Limitations and recommendations

The current research has its limitations due to certain factors that are namely, (a) data was gathered from communication students and not professional journalists, which can have different outcomes regarding results; (b) data were obtained from one country and two major cities which diminishes the generalizability of the findings; (c) due to scarcity of the theoretical frameworks that fit the current study, certain operational limits faced the researchers that hindered its process of conduct. Noting these limitations, scholars interested in the current topic can tackle the issue from different aspects such as the deployment of surveys to provide theoretical settings and examine causality, longitudinal data collection to assess changes over time, comparative studies from neighboring countries to provide a better understanding on cultural dimensions, and mixed-method studies where through triangulation, methods and theories can be drawn to better incorporate the psychological aspects linked to citizen journalism and their subsequent societal effects. Lastly, future studies can hold interviews with citizen journalists to better understand their views and perspectives regarding psychological and social influences and aspects of their news-generation efforts.

## Data Availability

The data used to support the findings of this study are included in the article.
